# Analysis of Twenty-one Cases of Articular Rheumatism

**Published:** 1854-03

**Authors:** Austin Flint

**Affiliations:** Buffalo


### BUFFALO MEDICAL JOURNAL

AND

### MONTHLY REVIEW.

**VOL. 9.** MARCH, 1854. **NO. 10.**

### ORIGINAL COMMUNICATIONS.

### ART. I.—*Analysis of Twenty-one Cases of Articular Rheumatism.*

By AUSTIN FLINT, M. D.

I propose in this paper to give, as concisely as possible, the results of an analysis of twenty-one cases of articular rheumatism. Of this number I have histories recorded either at the bedside, or while the cases were under observation. The number is not large. It would have been considerably larger had I recorded the histories of all the cases that have fallen under my observation. The disease, however, is not of very frequent occurrence in this city, (Buffalo.) In proof of this statement, on referring to the register of the Buffalo Hospital of the Sisters of Charity, not a single case was admitted into that institution from January to November, 1849, the inmates in the medical wards, during that period, averaging from thirty to forty daily. Nor was even a single case admitted from Dec., 1849, to May, 1850. The same is true of the period from October, 1850, to October, 1851; the daily average number of inmates being much greater than before.

The infrequency of the disease in this region is interesting, considered in connection with the fact that the population of Buffalo embraces a very large proportion of young persons, and that the climate of this place is characterized by sudden and extreme changes of temperature. As respects climate, sudden alternations of temperature, and high winds form the most prominent features. Without being able to state the average dew point, the atmosphere is remarkably dry, notwithstanding its proximity to the lake.

Of the twenty-one cases which I shall proceed to analyze, in seventeen the disease was, moderately or otherwise, acute, and in the remaining four cases, sub-acute.

*Age.* The ages ascertained, respectively, were as follows:—22, 40, 33, 30, 21, 15, 18, 16, 22, 30, 30, 23, 32, 32, 26, 28, 24, 21; in all, nineteen cases.

Mean age, $25\frac{1}{9}$ years.

*Season.* The cases occurred in the following months:—Jan., 4 cases; Feb., 3 cases; March, 4 cases; April, 2 cases; May, 1 case; June, 1 case; July, 0 case; August, 1 case; Sept., 0 case; Oct., 3 cases; Nov., 1 case; Dec., 1 case.

*Previous Health.* Of ten cases in which the facts with respect to the occurrence of previous attacks of rheumatism were ascertained, in six the patients had had the disease. One patient had had two previous attacks, and one had had several; the remaining eight had previously had the disease but once. One patient had been subject to frequent attacks of neuralgia; one had suffered long and much from spinal irritation, and two had had intermittent fever. In one instance, the disease followed close on the steps of intermittent fever; and in one, dysentery had shortly preceded it.

*Mode of Attack.* The invasion was sudden in nine cases, and in four of these the attack took place in the night time. In seven cases the disease was gradually developed.

*Sex.* Eighteen were males, and three were females; the greater relative liability of the male sex to rheumatism being thus illustrated.

*Parts affected in combination and succession. Local symptoms.* Under this head the first points of inquiry are: Of the different joints of the body, which are most liable to be affected in rheumatism; and in what order do the joints severally rank as respects this liability? The facts bearing on these points of inquiry are as follows:—In the twenty-one cases, the knee, or knees, were affected in seventeen; the ankle joint in fifteen; the wrist in fourteen; the shoulder in eleven; the hip in nine; the elbow in eight; the phalangeal joints of hand in six; the phalangeal joints of foot in two, and the maxillary in one.

The ligaments of the cervical vertebræ were affected in two cases; of the back in five cases; of the dorsum of hand in one case, and of the foot in two cases.

A second point of inquiry is, what joints were simultaneously affected at the time of the attack? Both knees were affected in four cases; both ankles in two cases. The following combinations were each (with one exception) exemplified in a single case: Both wrists; hips, knees, ankles and wrists; ankles and toes of both feet; left hip and left knee; elbow and shoulder joints; both knees and both ankles; right knee and right ankle; left hip, knee and ankle; both shoulders; left ankle and left wrist (two cases); both ankles, knees and hips.

An examination of the combinations of joints simultaneously affected is interesting with reference to the *law of parallelism.* Rheumatism is one of the *symmetrical* diseases. Common observation shows that corresponding parts on the two sides of the body are apt to be attacked at the same time. Does this occur sufficiently often, or, in other words, are the exceptions so infrequent, that it may with propriety be regarded as a law of the disease? This question has bearings beyond simply the natural history of rheumatism. The existence of such a law is of importance in its relations to the pathology of the disease. It shows that the local manifestations of rheumatism involve, in the first place, an internal determining morbid condition; and that parts are affected, mainly or solely, in consequence of this ulterior condition, irrespective of external local causes. And, in the second place, it certainly favors the doctrine that this internal morbid condition, thus determining the situation of the local rheumatic inflammations, pertains to the blood. To suppose that there is a kind of elective affinity between the structures attacked and a morbid matter brought to them by the blood, is to account most rationally for the fact that a disease affects similarly corresponding parts of the two symmetrical halves of the body. And in support of this hypothesis, it may undoubtedly be affirmed that most symmetrical affections, irrespective of the law of parallelism, appear to be blood diseases. It will at once occur to the mind of the reader that were we authorized to say that a well-marked law of parallelism sufficiently establishes the blood pathology of a disease, it may be applied to settle the question of the pathological character of some affections in which other evidence of their dependence on a morbid condition of the blood is wanting.

Reverting to the combinations already given of joints simultaneously affected, in thirteen of the eighteen instances the same joints on the two sides of the body are associated. Of the five instances in which this was not the fact, the left ankle and wrist were here simultaneously affected in two; and in the remaining three instances the knee and ankle, and the hip and knee on one side were affected in combination. These are not properly exceptions to the law of parallelism; they are simply negative instances in this point of view. True, exceptions would be instances in which different joints on the two sides of the body are affected in combination. There are no such instances in the combinations already given, and we may, therefore, conclude that, thus far in the analysis, the law referred to is sustained by the majority of the instances in which different joints were attacked, simultaneously, and no positive exceptions are found. It may be remarked here, that in examining with reference to the point under analysis, it will be fair to consider instances in which corresponding joints of the upper and lower extremity are affected in combination, as certainly not deviating from, if, indeed, they are not exemplifications of a law of parallelism. The knee and elbow, the wrist and ankles, the hip and shoulders are analogues; and were, for example, the wrist on one side, and the ankle on the other side to be affected in combination, it would not form an exception to the rule of symmetry. This consideration has no reference to the facts already presented, but it may, perhaps, be applicable to those which are now to follow.

What joints were affected in combination subsequently to the date of the attack? The following are the enumerations developed by an examination of the histories with reference to this inquiry:

*Parallel joints affected.*

| | |
|---|---|
| Both knees, | 4 cases. |
| Both wrists, | 5 " |
| Both shoulders, | 4 " |
| Both elbows, | 3 " |
| Both hips, | 1 case. |
| Both ankles, | 3 cases. |
| Great toes, | 1 case. |
| Both knees and ankles, | 1 " |
| Elbows and wrists, | 2 cases. |
| Shoulders, elbows and hips, | 1 case. |
| Total, | 25 |

*Single joints affected.*

| | |
|---|---|
| One knee, . . . . . . | 3 cases |
| One wrist, . . . . . . . | 14 " |
| One shoulder, . . . . . . | 6 " |
| One elbow, . . . . . . . | 3 " |
| One hip, . . . . . . . | 3 " |
| One ankle, . . . . . . . | 3 " |
| Total, . . . . | 32 |

*Different joints affected on same side, and joints not corresponding on the two sides.*

| | |
|---|---|
| Right wrist, elbow and shoulder, . . . . | 1 case. |
| Wrist and finger joints, . . . . . . . . | 1 " |
| Finger joints of one hand, . . . . . . . | 7 cases. |
| Left ankle and right hip, . . . . . . . | 1 case. |
| All joints of right arm and mostly all joints of left arm, | 1 " |
| Right hip, knee and ankle, . . . . . . . | 1 " |
| Left wrist and left ankle, . . . . . . . | 1 " |
| Total, . . . . . . . | 13 |

The foregoing results show the number of instances in which a single joint was affected to be in the majority; next in the order of frequency, parallel joints were affected; and in a much less number of instances different joints on the same side, or not corresponding on the two sides, were attacked in combination.

Directing attention to the evidence for or against the law of parallelism, we find in the first class of combinations the positive exemplifications of this law. In the second division, viz., in which single joints were affected, the evidence is, as has been seen, negative. We are to look in the third class for the exceptions to the law. And the exceptions are instances in which different joints on the two sides of the body, not analogues, were affected in combination. There is but one instance of that character. In that instance the left ankle and the right hip were simultaneously affected. In no instance were joints different, but analogues, affected in combination. The consideration, therefore, with reference to the latter point, which was premised, is not applicable to the facts developed by this analysis. The other instances in this division, viz., those in which different joints on the same side were affected in combination, are to be regarded in the same light as those in which single joints were affected. They are negative as respects their bearing on the law of parallelism. *In only a single instance, in all the combinations of joints simultaneously affected, both at the time of the attack, and subsequently, during the progress of the disease, in the twenty-one cases, was there a positive confliction with the law of parallelism.*

To present the facts in another aspect, the whole number of instances in which the joints were affected, in the twenty-one cases, either singly, or in the different complications, is eighty-eight. So far as the results of this analysis go, therefore, the ratio of exceptions to the law of parallelism in rheumatism, is as 1 to 88.

It is interesting to notice that certain joints are specially disposed to be affected in combination on the two sides, *e. g.* the knee, while other joints are apt to be affected singly, *e. g.* the wrist; and the joint most apt to be affected singly, viz., the wrist, is one of the least liable to be affected double.

With respect to the local symptoms, in no instance were swelling or redness present when the hip and shoulders were affected. These symptoms were generally, but not uniformly present when the knee, ankle, wrist and finger joints were the seat of the manifestations of the disease. Increased heat was sometimes present when the hip and shoulder joints were affected.

*Heart complications.* In fifteen cases the existence, or non-existence of heart complications, was determined. Of these fifteen cases, the heart was affected in six, and unaffected in nine. The evidences and history of heart affection in the six cases, respectively, are as follows:

Case I.—On the fifth day after my attendance commenced, and the eighth day from the date of the attack, a soft, systolic endocardial murmur was observed. The action of the heart was not increased. The febrile movement had subsided. Some pain was felt in and below the precordia, and there was tenderness on pressure in the same situations. The patient was convalescent on the seventh day from the date of my attendance, and slowly recovered. The disease occurred in March, 1852. During the subsequent winter he had a second attack of rheumatism, having never had the disease before the preceding March. He was not under my observation during the second attack. He recovered from it, but the state of the heart, and of the general health, is not known. Age of patient, 15 years.

Case II.—On the seventh day from the date of the attack, a loud and somewhat rough systolic endocardial murmur existed. At this date the patient came under my charge, and the previous existence, or non-existence, of the murmur had not been ascertained. The murmur was heard at, and above the base of the heart, not at the apex. There were no signs of enlargement. Patient had had an attack of rheumatism ten years before. Age of patient, 16 years. The case was pretty severe and protracted.

Case III.—Patient came under observation on the sixth day after the attack, and on the ninth day a soft, systolic endocardial murmur was observed. The impulse was normal. The murmur diminished and disappeared after convalescence was established. He had slight pain in precordia on deep inspiration. The case was mild, and brief in duration. Subsequent history of patient unknown. Had not had the disease before. Age, 22 years.

Case IV.—On admission into hospital about a week after the date of the attack, he had a soft, systolic endocardial murmur, low in pitch, maximum of sound at the apex, the heart's impulse somewhat increased. There was pain in precordia on deep inspiration, and on acts of coughing. The abnormal impulse ceased, but the murmur continued till his discharge. The case was mild and brief. It does not appear that he had had the disease before. Age, 17 years.

Case V.—Admitted on the ninth day of the disease, and on the twelfth day was observed a systolic endocardial murmur, the maximum between the second and third ribs, high in pitch. The area of flatness of percussion sound in precordia was increased, and there was no apex impulse. The disease was mild. Patient was convalescent on the twenty-third day. History after discharge unknown. Age, 32.

Case VI.—On the sixth day from the date of the attack, a soft, systolic murmur was observed, the maximum between second and third ribs. Action of heart not increased. No precordial pain. The disease was mild. Patient was discharged on the eleventh day from the date of the attack. Endocardial murmur at the time of discharge faint. Subsequent history not known. Age, 26.

In the foregoing six cases, as will be noticed, the evidences of endocarditis alone, were present in five, and of endo and pericarditis in but one.

The rheumatism was mild in all cases but one, and in the latter tolerably severe.

The endocardial murmur was produced at the mitral orifice in one case, and at the aortic orifice in four cases.

Precordial pain existed in four cases; it did not exist in one case, and in one case nothing is noted on this point. In no instance was the precordial pain severe, or prominent as a symptom.

In each case convalescence took place without leaving any consciousness, on the part of the patient, of a heart affection. In one instance the endocardial murmur disappeared, in one instance it became faint, and in the other cases it persisted without any notable change.

The maximum of the duration of the rheumatism in the cases complicated with heart affection, was twenty-six days. The next longest duration was twenty-three days; the next, twenty-one days, and the next, twelve days. The minimum of duration was seven days. The mean duration was sixteen $\frac{2}{6}$ days.

Comparing the mean duration of these cases with the mean duration of all the cases, the latter, as will be presently seen, was seventeen $\frac{8}{17}$ days. So that the mean duration of the cases with heart complication is somewhat less than the mean duration of all the cases! The duration in the facts just presented is considered to embrace the period between the date of the attack, and the date of convalescence. Calculating from the date of attack to the time of discharge, and the results in the two groups of cases are as follows: in the cases with heart complication, mean duration, twenty-six days; do., in all the cases, twenty-nine days! This shows, as in the former comparison, a shorter duration of the cases in which heart affection exists! So far as deductions from these facts are admissible, it is evident that heart complication exerts no influence to protract either convalescence, or apparent recovery from the disease.

*Pain, and other symptoms pertaining to Nervous System.* In all the cases, pain in the affected joints was more or less prominent. In some it was severe, in others slight. In several instances it is noted that more pain was experienced during the night, than in the daytime. Shooting neuralgic pains were noted in one case. Hysterical attacks, accompanied by neuralgic pains in the abdomen and chest, occurred near the time of convalescence in one case. Tenderness over the spinal column is noted in two cases.

*Disorder of Digestive System.* The appetite was generally either lost or impaired, but was sometimes preserved when the disease was subacute, or moderately acute.

The tongue was usually more or less coated.

Constipation was present in the great majority of cases. Diarrhœa, occurring spontaneously in the course of the disease, is noted but in one case.

Thirst was frequently present.

*State of Urine and Perspirations.* The urine deposited, on cooling, the urate of ammonia in fourteen cases. In the remainder of the cases the state of the urine was not noted, or noted too imperfectly to determine the non-existence of this deposit throughout the disease. The uratic deposit was observed to disappear in several instances, at or near the time of convalescence, and after a marked improvement in the symptoms had taken place. Its continuance after convalescence was established, or after marked improvement, is not noted in a single case.

The sp. gr. taken in one case, once daily, for nine consecutive days, averaged 1.015, varying at different times from 1.012 to 1.017. In another case the sp. gr. at two observations was found to be 1.030, and 1.028.

The urine was albuminous in one case, and the histories contain information on that point in this case only.

Sweating, more or less frequently, and profuse, occurred in seventeen cases; and the absence of perspirations is not noted in the history of a single case. The sweating was slight, and persisted but for a short time in a case characterized by diarrhœa, this being the only case in which the latter symptom occurred. In this case the perspirations did not take place till after the diarrhœa had ceased.

In almost every instance in which perspirations are noted to have occurred, they were either confined to the night, or were more profuse at that time.

It does not appear that the sweating was ever followed by relief of the affection of the joints.

*Pulmonary Symptoms.* The absence of pulmonary symptoms is the chief fact pertaining to this head. In two cases only is it noted that any cough and expectoration were present, and in them these symptoms were slight.

### *Duration.*

TABLE SHOWING DURATION OF DISEASE IN EIGHTEEN CASES.

| | No. 1. DAYS. | No. 2. DAYS. | No. 3. DAYS. | No. 4. DAYS. | No. 5. DAYS. | No. 6. DAYS. | No. 7. DAYS. | No. 8. DAYS. | No. 9. DAYS. | No. 10. DAYS. | No. 11. DAYS. | No. 12. DAYS. | No. 13. DAYS. | No. 14. DAYS. | No. 15. DAYS. | No. 16. DAYS. | No. 17. DAYS. | No. 18. DAYS. |
|---|---|---|---|---|---|---|---|---|---|---|---|---|---|---|---|---|---|---|
| From attack to convalescence,.. | 5 | 11 | 16 | 7 | 7 | 31 | 7 | 8 | 26 | 12 | 32 | 12 | 23 | 23 | 21 | 9 | 0 | 47 |
| From attack to discharge,....... | 11 | 0 | 30 | 0 | 7 | 40 | 7 | 18 | 44 | 17 | 46 | 21 | 78 | 0 | 0 | 12 | 57 | 0 |

Mean duration to convalescence, $17\frac{8}{17}$ days.

Mean duration to discharge, $29\frac{1}{13}$ days.

In no instance did the disease prove fatal.

In no instance did the acute eventuate in the chronic form of the disease.

In two instances, only, did apparent convalescence take place, and a recurrence of the severity of the symptoms follow.

*Causative influences.* In the histories of 14 cases nothing is contained relative to causation, except in two instances it is noted that the patients were free livers.

In seven cases the patients attributed the disease to exposure to cold.

In one case the patient had been exposed to over-exertion, and mental anxiety prior to the attack.

In one case the rheumatism occurred as a sequel of dysentery; and in one case it followed intermittent fever.

*Treatment.* In fifteen of the cases the treatment was mixed, *i. e.*, it consisted of different remedies. Of the remaining six cases, in two the treatment consisted solely, or almost exclusively, of the nitrate of potash; in two, solely of colchicum and bleeding, and in one case the account of treatment is incomplete.

The following remedies were prescribed, severally, in the number of cases appended:

| | | |
|---|---|---|
| Colchicum, | . . . . . . . . . . | 3 cases. |
| Nitrate of Potash, | . . . . . . . . | 9 " |
| Bleeding, | . . . . . . . . . . | 2 " |
| Mercury | . . . . . . . . . . | 8 " |

| | |
|---|---|
| Opium, (in form of gum, sulphate of morphia, or Dover's powder,) | 14 cases. |
| Citric Acid, | 5 " |
| Sulphate of Quinia, | 7 " |
| Iodide of Potash, | 7 " |
| Digitalis, | 1 case. |
| Lemon Juice, | 3 cases. |
| Liquor Potassæ, | 2 " |
| Phosphate of Ammonia, | 1 case. |
| Vesication, | Several cases. |

With respect to the apparent effects of these remedies, an interrogation of the histories develops the following facts and conclusions:

*Colchicum.* 1. This remedy was carried to the extent of vomiting and purging, with, apparently, marked relief, but the symptoms returned in three days with more severity than before. Duration of the disease not determinable. Colchicum and bleeding were the only remedies employed in this case.

2. Colchicum was carried to vomiting and purging, with apparent relief. Colchicum and bleeding were the only remedies employed. Duration of the disease to convalescence, five, and to discharge, eleven days.

3. Twenty drops of the tincture of colchicum were given every four hours. The apparent effect does not appear from the history.

4. Colchicum was carried to vomiting and purging. No relief of symptoms. Under its operation an endocardial murmur was first discovered.

*Inference.* Colchicum, carried to vomiting and purging, sometimes produces apparently marked relief, and sometimes none whatever. The relief which follows is not always permanent. Under the operation of this remedy endocarditis may become developed.

*Citric Acid.* Two cases were treated exclusively with citric acid.

1. Citric acid was given in doses increased to an ounce daily. Patient admitted into hospital three weeks after the date of the attack. Had taken, before his admission, only a purgative. Convalescent in eleven days, and discharged twenty-seven days after admission.

2. Citric acid in doses increased from two drachms to an ounce daily; and on one day two ounces were taken. Patient entered third day after date of attack, having had no medicine prior to his admission. Convalescent on the twelfth day after the attack, and discharged, quite well, on the twenty-first day.

Citric acid entered somewhat into the treatment of several other cases, but it is impossible to determine what effect it may have produced on the course of the disease.

In both the cases treated with citric acid, exclusively, the duration of the disease to convalescence and discharge was less than the average duration in all the cases.

*Nitrate of Potash.* One case was treated exclusively with this remedy. The patient entered two weeks after the date of the attack. He was convalescent and able to walk about twenty-three days after admission, and discharged shortly afterward.

Facts pertaining to the cases in which this remedy was employed in addition to other remedies, are as follows:

1. Symptoms relieved the day after the remedy was prescribed, but remainder of history defective.

2. Effect does not distinctly appear in the history, but it is presumed not to have been altogether satisfactory, as, after two days, the sulphate of quinia was added.

3. Continued through the disease in addition to the proto-iodide of mercury, and lemon juice. Convalescence in seven days.

4. Commenced on the fourth day in dose of two drachms daily. This constituted the treatment, with the exception of the sulphate of morphia at night, and the free use of lemonade. Patient had previously been purged with the sulphate of magnesia, and had taken the sulphate of morphia. Convalescence in seven days.

5. Entered six days after the date of the attack. Nitrate of potash, an ounce daily. Endocarditis developed four days after admission. He was then mercurialized. Convalescence in twenty-five days, and discharged thirty-eight days after admission.

6. Nitrate of potash an ounce daily, with calomel a grain hourly, endocarditis existing. Under this treatment daily improvement. Convalescence five days after admission, twelve days after the date of the attack.

7. Nitrate of potash and mercury continued to ptyalism. Case complicated with endocarditis. This treatment commenced on the fifth day after admission, and fourteenth day after date of attack. Previously treated with colchicum, under which endocarditis became developed. Under the use of the nitrate of potash and mercury, rapid improvement. Convalescence fourteen days after admission, and discharged in twenty-three days. This paient had as hort relapse after appearing to be convalescent.

8. Twenty-four days after the date of the attack, nitrate of potash half an ounce daily. Previously had had this remedy in small doses in conjunction with colchicum. Continued in the quantity above stated for ten days. Under its use the urine became clear, and the symptoms relieved. The urine was increased in quantity. After the continuance of the remedy for ten days, the sulphate of quinia, in doses of five grains three times daily, was substituted.

*Conclusions.* *(a.)* Duration of disease not always abridged by this remedy; see No. 5, in foregoing list, in which duration was considerably longer than the average.

*(b.)* In some instances relief follows the use of the remedy, and appears to be an effect of it. In other cases no decided relief follows, even when the remedy is continued for several days.

*(c.)* Endocarditis may be developed while the patient is under the use of this remedy. See case No. 5.

*(d.)* No unpleasant effects are noted, the remedy having been given, in several instances, in doses of an ounce in the twenty-four hours.

*(e.)* That the remedy possesses much power to control the disease is not apparent from the analysis of these few cases.

*Bleeding.* 1. Colchicum had been given, with transient relief, in this case. Four days afterward, the symptoms having returned with increased severity, bleeding was practiced, there being but moderate febrile movement, and the pulse not hard. Relief followed; but on the day but one afterward, the symptoms regained their former severity.

2. Bleeding was practiced on the evening of the second day. No improvement of the symptoms followed. On the next day colchicum carried to the extent of vomiting and purging. Relief followed this remedy. On the next day another bleeding, after which the relief became more decided. Febrile movement nearly gone, and from that date convalescence proceeded rapidly.

*Summary.* In the first case, transient relief only appears to have followed the bleeding. In the second the remedy appeared to contribute to the abatement of the disease, and the relief was permanent.

*Mercury.* 1. The proto-iodide of mercury was given, in doses of one grain three times daily, for several days, but not carried to ptyalism. The use of mercury was preceded by the nitrate of potash and lemon juice. The precordia was blistered. Endocardial murmur existed. Convalescence in seven days.

2. Calomel, grs. ii, and opium, grs. ii, every four or six hours, was continued till eight doses were taken. Then suspended on account of the existence of diarrhœa. Case accompanied by endocardial murmur.

3. Patient had taken calomel for six days, in small doses, before admission into hospital. On the fourth day after admission, the existence of endocarditis being determined, he was again placed under the use of calomel, and it was continued till ptyalism was induced. Prior to the discovery of endocarditis, after admission, the nitrate of potash had been given. Convalescence in twenty-six days, and recovery from the rheumatism after thirty-eight days.

4. Endocarditis existed in this case. Calomel given at once, in doses of one grain hourly. Also inunction with mercurial ointment, and blister to precordia. Ptyalism speedily induced. Convalescence in five days, and the patient was quite well in ten days after admission.

Had been affected with the disease for about a week prior to his admission, but had kept the bed for two days only.

5. Citric acid was the only remedy given for twelve days. Pericarditis then suspected, and calomel, in doses of a grain, with Dover's powder, given three times daily. Continued till ptyalism was induced.

Intermittent fever supervened. Patient discharged seventy-eight days after date of attack, quite well.

6. Endocarditis determined on the fifth day after admission, and fourteenth after the date of attack. Colchicum had been the remedy employed up to the discovery of endocarditis. Calomel was then prescribed in conjunction with digitalis, squill, and Dover's powder; and a blister applied to the precordia. Mercury was carried to mild ptyalism. Convalescence on the fourteenth day after admission, and twenty-third after the date of attack. Had a brief relapse, but was shortly afterward discharged, quite well.

7. On admission, tenth day after the date of attack, calomel, one grain, with Dover's powder, ten grains, twice daily, was prescribed, and continued for four days. Citric acid was also given. At the end of four days all treatment was suspended. Convalescence on the eleventh day after admission, and the twenty-third from the date of attack.

8. On admission, fourth day of disease, calomel, one grain, with Dover's powder, ten grains, was given twice daily. Under this treatment the evidences of endocarditis appeared the second day after admission, and the sixth day of the disease. Calomel, one grain, and Dover's powder, three grains, three times daily, were prescribed; a blister to precordia, and the nitrate of potash, three drachms in the twenty-four hours. Ptyalism was not induced. Convalescence on the fifth day after admission, and the ninth after date of attack. Patient discharged on the eighth day after admission, and the twelfth day after the date of the attack. Bellows sound scarcely appreciable at the time of the discharge.*

*Summary.* In six of the foregoing eight cases, endocarditis was presumed to exist from the presence of the bellows murmur; and in one of the two remaining cases, pericarditis was suspected.

In all the cases in which heart complication existed, mercury was prescribed.

In four of the cases ptyalism was induced.

Endocarditis became developed in one case in which mercury had been given for six days; and in another case, in which it had been given in doses of a grain twice daily for two days.

With the exception of one case, mercury was not prescribed (save as a purgative) unless heart complication existed, or its existence was suspected.

It has been already seen that the average duration of the cases with heart complication was less than the average duration of all the cases. To what extent, if to any, this fact is due to the mercurial treatment, cannot be settled by the data embraced in the present collection of observations.

*Opium.* 1. Dover's powder was given with apparent relief.

2. Opium, one grain repeated in three, four, or six hours, appeared to afford marked relief of suffering.

3. Sulphate of morphia, one-fourth of a grain, repeated every four or six hours. Apparent effect not noted.

4. Opium, and the sulphate of morphia, were given throughout the disease with apparently marked relief of suffering, but with no apparent effect on the progress of the disease.

5. Opium, with the phosphate of ammonia, constituted the chief remedy. The disease supervened on dysentery. Convalescence on tenth day, and quite well on eighteenth day.

---

* It is worthy of notice that in this case, in which the physical sign of endocardial exudation diminished in a notable degree, ptyalism was not induced, and a small quantity of mercury only was given.

6, 7, and 8. Dover's powder in combination with calomel.

9. Dover's powder, ten grains, and calomel, one grain, twice daily, continued for four days. No other treatment, save sol. citric acid for drink. Convalescence on the eleventh day, and discharged on the twenty-first day.

10. Dover's powder, ten grains, and calomel, one grain, twice daily, for two days. The existence of endocarditis was then determined. Afterward Dover's powder was continued in combination with mercury.

11. Sulphate of morphia, half a grain, at night. Relief of symptoms followed, but no apparent effect on the progress of the disease.

*Conclusion.* Opiates afforded relief of suffering, but these cases furnish no evidence of aught beyond a palliative effect.

Endocarditis became developed in one case in which Dover's powder, ten grains, twice daily, had been continued for two days.

*Sulphate of Quinia.* This remedy was given in several cases, in doses of one or two grains, repeated three or four times daily, at or near the time of convalescence. The instances in which it was given in larger doses, during the progress of disease, are as follows:

1. On the fifth day the iodide of potash was suspended, and the sulphate of quinia, twelve grains in the twenty-four hours, substituted. It was continued in this quantity for several days, but the patient not improving so well under its use as before, the iodide of potash was resumed, and the sulphate of quinia discontinued.

2. At the tenth day of the disease, the sulphate of quinia, four grains, three times daily, was prescribed, continued for three days, and then discontinued, no apparent effect being produced.

3. Sulphate of quinia, five grains, three times daily, was given thirty-one days after the date of the attack, the treatment preceding having consisted of the nitrate of potash. The remedy was continued for ten days, and the dose increased to eight grains, three times daily. It was continued in these doses for seven days. The improvement was very gradual, and at the end of the period just named, the iodide of potash was substituted.

*Conclusions.* No evidence is afforded by these few cases of the efficacy of the sulphate of quinia in controlling the morbid processes upon which rheumatism depends. The effect was wholly negative.

*Iodide of Potash.* 1. Six days after the date of the attack, the iodide of potash was prescribed. Commencing with twelve grains in the twenty-four hours, the quantity was increased to thirty-two grains. It was continued in the quantity last named for four days. In this period moderate improvement had taken place. The sulphate of quinia was substituted, but the patient appearing not to improve under the latter treatment, the iodide of potash was again resumed, and the sulphate of quinia discontinued. Commencing with twenty grains, the quantity was increased to sixty grains in the twenty-four hours. Under this treatment convalescence gradually took place. The patient was sufficiently restored to walk about with crutches a month after the date of the attack.

2. The iodide of potash was given during the progress of the disease, in doses of five grains, three times daily. The nitrate of potash, and the sulphate of quinia had previously been employed. The patient improved about equally under each of these remedies.

In addition to the foregoing, the iodide of potash was prescribed in two of the cases, but with what effect does not appear from the histories.

It is evident that these few observations furnish no positive evidence of a controlling efficacy in the iodide of potash.

*Lemon Juice.* This was given as a remedy in three of the cases. In one case it had been given freely before the patient came under my charge. It was continued till diarrhœa occurred, and the stomach revolted against its farther use. No apparent effect on the progress of the disease was produced by it.

In the other cases it was not given in large quantity, and it was associated with other remedies, so that its effect could not be estimated.

*Liquor Potassæ.* This remedy was prescribed in two of the cases. In one no apparent effect was produced; nor in the other case could any remedial influence be fairly attributed to it, the improvement taking place very slowly.

*Concluding Remarks.* The results developed by the analysis of the few cases contained in this collection are of importance only as a small contribution toward the accumulation of facts by which it is to be hoped, the merits real and relative, of the several remedies that have been noticed, may be determined. As the true point of departure for studying the effects of any, or all remedies, our science lacks here, as with reference to most other diseases, knowledge of the average duration, etc., of a series of cases in which the disease was allowed to pursue its course undisturbed by medicinal interference. This knowledge cannot be voluntarily and deliberately acquired. Facts bearing thereon can only be obtained slowly, as chance supplies them.

And, at the present moment, we cannot answer the question, what are the intrinsic tendencies of articular rheumatism as respects its continuance, its complications, and remote consequences in the organism? Were we able to answer this question by an appeal to facts, we should then have a criterion by which to estimate the favorable or unfavorable influences of different measures of treatment pursued in a series of cases. As it is, in bringing statistical information to bear on the therapeutics of the disease, we can only study the immediate apparent effects of different remedies, and institute comparisons, in this point of view, and also with reference to the duration of the disease, etc., in different series of cases treated by different methods.

So far as the few observations go which have been presented in this paper, they lead to a distrust of the efficacy of the several remedies employed, rather than tend to increase confidence in our ability to control, by means of them, the progress of the disease. Some of the remedies appear to possess more or less palliative power. This is true of colchicum, bleeding, and opium; but as respects the duration of the disease, it was in some cases short, and in other cases protracted, under different remedies. This being so, it is, perhaps, more philosophical to attribute these differences to variations in the tendencies of the disease, in different cases, rather than to the agency of the remedies used.

It is to be observed that under the use of several remedies supposed to possess more or less remedial efficacy in this disease, the complication most to be apprehended, viz., heart affection, became developed. This was true of colchicum, the nitrate of potash, calomel, and opium.

Inasmuch as the purpose of this paper is simply to give the facts elicited, by the analysis of the few cases, the histories of which I have recorded, discussion of any of the various questions which suggest themselves relating to therapeutics, as well as other branches of the subject, would be out of place.

BUFFALO, February, 1854.

